# Awareness of Amblyopia Among the Population of Jazan, Saudi Arabia: A Cross-Sectional Survey

**DOI:** 10.7759/cureus.73346

**Published:** 2024-11-09

**Authors:** Ahmed A Bahri, Essam A Alhazmi, Yazeed A Hamzi, Faisal H Abusageah, Bander H Ageeli, Ali M Shawish, Weaam A Najmi, Khaled S Alghamdi, Rana I Abuhadi, Fatimah A Alahdal, Yara M Adawi, Zaher T Hakami, Fouad I Hakami, Linan M Khormi

**Affiliations:** 1 Preventive Medicine, Jazan University, Jazan, SAU; 2 College of Medicine, Jazan University, Jazan, SAU; 3 College of Medicine, Albaha University, Albaha, SAU

**Keywords:** amblyopia, awareness, cross-sectional study, early detection, health education, jazan, lazy eye, public health, saudi arabia, visual impairment

## Abstract

Background: Amblyopia, or "lazy eye," is a visual disorder that often goes undiagnosed, especially in its early stages. Early detection and treatment are critical to preventing long-term visual impairment. This study aimed to assess the awareness of amblyopia among the population of Jazan, Saudi Arabia.

Methods: A cross-sectional survey was conducted from May to October 2024 to evaluate amblyopia awareness in Jazan. The study utilized a random sampling technique and data were collected via an online questionnaire. A total of 405 participants were included in the analysis, with the sample size determined using the Raosoft sample size calculator (Raosoft, Inc., Seattle, Washington, United States), ensuring a 95% confidence interval and a 5% margin of error.

Results: The findings revealed that 68.6% (n=278) of participants had heard of amblyopia, with a significant percentage of respondents (40.7%, n=165) reporting no family history of eye conditions. Awareness varied significantly across educational levels, with 74.1% (n=300) of college-educated participants being familiar with the condition. Urban residents (45.7%, n=185) showed higher awareness than rural residents (54.3%, n=220). Participants also demonstrated limited knowledge about the visibility of amblyopia without examination, with 40.0% (n=162) unsure if it could be detected visually. Additionally, 35.3% (n=143) did not know whether a general practitioner could diagnose the condition.

Conclusion: The study indicates a significant gap in amblyopia awareness, particularly among rural populations and individuals with lower educational attainment. Public health campaigns and early screening programs are essential to improve awareness and for early diagnosis. Additionally, enhancing the role of healthcare providers in amblyopia education and detection is crucial for reducing the burden of untreated amblyopia in Jazan.

## Introduction

Amblyopia, often referred to as "lazy eye," is a visual impairment that occurs when the brain favors one eye over the other, leading to reduced vision in the weaker eye. This condition typically develops during childhood and, if left untreated, can lead to permanent vision loss in the affected eye [1-5]. It is one of the most common causes of visual impairment in children worldwide, affecting approximately 2-3% of the population. Early diagnosis and intervention are crucial for effective treatment, as the visual system is most responsive to correction during the early years of life [2,6].

The prevalence of amblyopia varies across different populations and regions. It is most commonly caused by strabismus (misalignment of the eyes), refractive errors such as uncorrected farsightedness or nearsightedness, and deprivation of visual input (e.g., due to cataracts or ptosis). Other risk factors include a family history of amblyopia, premature birth, and certain systemic conditions such as cerebral palsy or Down syndrome [3-8]. The condition is more commonly diagnosed in children, but if not addressed by the age of seven or eight years, the brain’s ability to process visual information from the weaker eye becomes less flexible, making treatment more difficult.

Awareness of amblyopia among parents, caregivers, and healthcare providers plays a crucial role in early detection and intervention. Early screening for amblyopia is recommended during pediatric health visits, particularly before the child enters school [5-10]. Despite the importance of early intervention, research indicates that public awareness of the condition is often limited. Many individuals are unaware that amblyopia is treatable if identified early, and some may not recognize the symptoms or understand the potential long-term consequences of untreated amblyopia. This lack of awareness can delay diagnosis and treatment, resulting in poorer visual outcomes.

In Saudi Arabia, there is a growing need for awareness campaigns and educational initiatives to inform the public about amblyopia. Despite improvements in healthcare access and the availability of screening programs, limited data exist regarding the general population's knowledge of the condition. Understanding the level of awareness of amblyopia in the region is essential to inform health promotion strategies and guide policy development. This study aims to fill this gap by assessing the awareness of amblyopia among the population of Jazan, Saudi Arabia, and exploring the factors that contribute to knowledge or lack thereof.

## Materials and methods

Study design

A cross-sectional study was conducted to evaluate the awareness of amblyopia among the population of Jazan, Saudi Arabia. The study was carried out between May 2024 and October 2024. This design was chosen to assess the knowledge and perceptions of amblyopia in a representative sample of the population at a single point in time.

Sample size calculation

The sample size was determined using the Raosoft sample size calculator (Raosoft, Inc., Seattle, Washington, United States, raosoft.com), which recommended a total of 377 participants to achieve a 95% confidence interval with a 5% margin of error. To account for any non-responses or incomplete surveys, a total of 405 participants were targeted to ensure statistical validity.

Sampling technique and inclusion criteria

A random sampling method was used to select participants for this study. The population of Jazan was divided into subgroups based on age, gender, and other demographic factors, ensuring that the sample was representative of the larger population. Participants were randomly selected within these subgroups to minimize selection bias and ensure diversity in the sample. Participants were eligible for inclusion in the study if they were residents of Jazan and were 18 years of age or older. Those who did not complete the survey or provided incomplete responses were excluded from the analysis.

Ethical considerations

The study was approved by the institutional review board (IRB) of Jazan University, Jazan (approval number: 46/02/1155). All participants were informed about the purpose of the study and gave their informed consent before participating. Confidentiality was maintained throughout the study, and no personal identifying information was collected. Participants had the right to withdraw from the survey at any time without consequence.

Data collection and analysis

Data were collected using an online survey (see Appendices) distributed through various digital platforms to reach a broad and diverse group of participants. The online survey was designed to be user-friendly, and participants were invited to complete it voluntarily. The questionnaire consisted of multiple-choice and Likert-scale questions, which assessed participants’ demographic characteristics, knowledge of amblyopia, as well as awareness regarding its causes, symptoms, and treatment methods. Data were analyzed using IBM SPSS Statistics for Windows, Version 26, (Released 2019; IBM Corp., Armonk, New York, United States). Descriptive statistics were calculated to summarize the demographic characteristics of the sample and participants' awareness of amblyopia. Frequencies (n) and percentages (%) were reported for categorical variables.

## Results

Demographic characteristics

A total of 405 participants were included in the study. Among them, 126 (31.1%) were aged 18-25 years, 190 (46.9%) were aged 26-40 years, and 89 (22.0%) were aged 41 years or older. Regarding gender, 209 (51.6%) were female, and 196 (48.4%) were male. In terms of marital status, 191 (47.2%) were married, and 214 (52.8%) were single. Concerning educational level, the majority, 300 (74.1%), had attended college, 44 (10.9%) had higher education, 57 (14.1%) had completed high school, and four (1.0%) had less than a high school education. As for residence, 185 (45.7%) participants lived in urban areas, while 220 (54.3%) resided in rural areas (Table 1).

**Table 1:** Demographic characteristics of study participants (N=405) Data are presented as frequency (n) and percentage (%). Percentages may not add up to 100 due to rounding.

Variable		Frequency (n)	Percentage (%)
Age group	18-25 years	126	31.1
	26-40 years	190	46.9
	41 years and above	89	22.0
Gender	Female	209	51.6
	Male	196	48.4
Marital status	Married	191	47.2
	Single	214	52.8
Educational level	College	300	74.1
	Higher education	44	10.9
	High school	57	14.1
	Less than high school	4	1.0
Place of residence	Urban (city)	185	45.7
	Rural (village)	220	54.3

Knowledge and awareness of lazy eye

Regarding family history of eye conditions, 240 (59.3%) participants reported having a family history of eye problems, while 165 (40.7%) reported no such history. When asked about their awareness of lazy eye, 278 (68.6%) participants confirmed they had heard of it, while 38 (9.4%) were unsure, and 89 (22.0%) had never heard of lazy eye. Concerning the visibility of lazy eye without examination, 162 (40.0%) were unsure, 160 (39.5%) believed it was not visible, and 83 (20.5%) thought it was visible without an examination (Table 2).

**Table 2:** Knowledge and awareness of lazy eye (N=405)

Variable		Frequency (n)	Percentage (%)
Family history of eye condition	No	165	40.7
	Yes	240	59.3
Awareness of lazy eye	Unaware	38	9.4
	Not heard	89	22.0
	Aware	278	68.6
Lazy eye visibility without examination	Uncertain	162	40.0
	Not visible	160	39.5
	Visible	83	20.5
Lazy eye diagnosis by general practitioner	Uncertain	143	35.3
	Not diagnosable	142	35.1
	Diagnosable	120	29.6
Lazy eye diagnosis exclusively by ophthalmologists	Uncertain	114	28.1
	Not diagnosable	57	14.1
	Diagnosable	234	57.8

In terms of diagnosis, 120 (29.6%) participants believed lazy eye could be diagnosed by a general practitioner, while 142 (35.1%) disagreed, and 143 (35.3%) were uncertain. In contrast, 234 (57.8%) participants believed that an ophthalmologist could exclusively diagnose lazy eye, while 57 (14.1%) disagreed, and 114 (28.1%) were unsure.

Treatment, causes, and information sources for lazy eye

When asked about the age group most affected by lazy eye, 18 (4.4%) participants believed it primarily affects adults, 283 (69.9%) stated it affects both children and adults, and 104 (25.7%) believed it affects children only. In terms of the recommended age for treatment, 169 (41.7%) participants indicated that there was no specific age period, 136 (33.6%) suggested treatment between three and nine years, 80 (19.8%) recommended before one year, and 20 (4.9%) suggested after 10 years (Table 3).

**Table 3:** Lazy eye treatment, causes, and information sources (N=405) Data are presented as frequency (n) and percentage (%). Percentages may not add up to 100 due to rounding.

Variable		Frequency (n)	Percentage (%)
Impact of lazy eye	Affects adults	18	4.4
	Affects both adults and children	283	69.9
	Affects children only	104	25.7
Recommended age for treatment of lazy eye	After 10 years	20	4.9
	Before 1 year	80	19.8
	Between 3 and 9 years	136	33.6
	No specific age period	169	41.7
Importance of vision screening before school	Uncertain	54	13.3
	Yes, important	341	84.2
	No, not important	10	2.5
Causes of lazy eye (amblyopia)	Nutritional deficiencies	124	30.6
	Trauma	111	27.4
	Cerebral palsy	75	18.5
	Refraction errors	120	29.6
	Genetic factors	257	63.5
	Down syndrome	72	17.8
	Maternal health issues	82	20.2
Treatment methods for lazy eye	No treatment	344	84.9
	Vision exercises	245	60.5
	Surgery	109	26.9
	Corrective glasses	251	62.0

Regarding the importance of vision screening before school, 341 (84.2%) participants believed it was important, 54 (13.3%) were unsure, and 10 (2.5%) felt it was not necessary. Concerning the causes of lazy eye, 257 (63.5%) participants believed genetic factors contributed, 120 (29.6%) pointed to refraction errors, 124 (30.6%) mentioned nutritional deficiencies, 111 (27.4%) identified trauma, 75 (18.5%) linked it to cerebral palsy, 72 (17.8%) associated it with Down syndrome, and 82 (20.2%) attributed it to maternal health issues.

Lastly, regarding treatment methods for lazy eye, 344 (84.9%) participants reported no treatment, 245 (60.5%) mentioned vision exercises, 251 (62.0%) indicated corrective glasses, and 109 (26.9%) cited surgery as a potential treatment option.

## Discussion

This study aimed to assess the awareness of amblyopia among the population of Jazan in an effort to understand the level of knowledge regarding this common yet often underdiagnosed condition. The findings highlight several key trends and implications for public health education and early intervention strategies.

The results of this study show that the majority of participants (68.6%) reported having heard of amblyopia, with significant differences in awareness based on educational level and residence location. While awareness was high among individuals with a college education (74.1%) and those residing in urban areas (45.7%), a notable proportion of participants, particularly in rural areas (54.3%), reported limited knowledge about amblyopia. This highlights a potential gap in health education efforts, as individuals with lower levels of education or those living in rural settings may not have access to the same resources or exposure to health information as those in more urbanized regions [2-7].

The finding that 40.7% of participants had no family history of eye conditions suggests that amblyopia may not be readily identified in families without a history of visual impairments. This is important because amblyopia can occur in individuals without any prior family history of eye disorders, indicating the need for universal screening, particularly during childhood [11,12].

Several factors likely contribute to the gaps in amblyopia awareness observed in this study. The relatively low level of knowledge about amblyopia’s visibility without examination (40.0% reporting “Don’t know”) and diagnosis by a general practitioner (35.3% reporting “Don’t know”) suggests a lack of clarity among the general population regarding how the condition can be detected. Amblyopia is not always apparent to the naked eye, and without proper screening, it may go unnoticed until the child is older, when treatment may be less effective. Moreover, the study found that many participants were unaware that amblyopia could be diagnosed and managed by a general practitioner or family doctor, indicating a need for better integration of amblyopia detection in general health assessments.

The data from this study underscore the importance of improving public education on amblyopia, particularly among parents and caregivers. Raising awareness about the significance of early screening and intervention is critical for reducing the prevalence of untreated amblyopia, as visual impairments in childhood can have lasting consequences on academic performance and quality of life. The high percentage of participants who reported learning about amblyopia from the Internet or social media (50.9%) points to the potential for leveraging digital platforms to disseminate information more effectively. Public health campaigns can be enhanced by focusing on platforms that are most widely used by the target audience, such as social media, and offering clear, accessible information about the importance of early vision screening [12-16].

Limitations

While the study provides valuable insights into the awareness of amblyopia in Jazan, several limitations must be acknowledged. First, the study used an online survey, which may have limited participation from certain groups, particularly those without access to the Internet or those with lower levels of digital literacy. Additionally, the self-reported nature of the survey may introduce response biases, such as social desirability bias or misunderstanding of questions. Future research could employ mixed-methods approaches, incorporating both qualitative interviews and quantitative surveys, to gain deeper insights into the factors influencing awareness and attitudes toward amblyopia. Another limitation is the cross-sectional nature of the study, which prevents the determination of causality between demographic factors and awareness of amblyopia. Longitudinal studies or studies using more robust sampling techniques could provide further evidence of the factors that contribute to amblyopia awareness and help assess the impact of educational interventions over time.

## Conclusions

In conclusion, this study highlights significant gaps in the awareness of amblyopia among the population of Jazan, particularly among individuals in rural areas and those with lower educational levels. Despite the relatively high overall awareness, many participants lacked knowledge about the condition's visibility, diagnosis, and treatment options. These findings underscore the urgent need for targeted public health interventions, such as awareness campaigns and early screening programs, to improve early detection and treatment of amblyopia. Furthermore, healthcare providers should be empowered to play a greater role in educating the public and ensuring the condition is diagnosed during routine health check-ups. By addressing these gaps, it will be possible to reduce the long-term visual impairment caused by amblyopia and improve the quality of life for affected individuals.

## Appendices

Survey on awareness and knowledge of amblyopia

Demographic Information 

1. Age: ☐ Under 18 ☐ 18-24 ☐ 25-34 ☐ 35-44 ☐ 45-54 ☐ 55+

2. Gender: ☐ Male ☐ Female

3. Education Level: ☐ Primary ☐ Secondary ☐ High school ☐ Undergraduate ☐ Graduate+

4. Children: ☐ Yes ☐ No

5. Family Eye Disease History: ☐ Yes ☐ No

Section A: General Knowledge of Amblyopia 

1. Have you heard of amblyopia (lazy eye)? ☐ Yes ☐ No

2. Source of Information: (Select all) ☐ Doctor ☐ Family/Friends ☐ Internet ☐ Social Media ☐ TV/Radio ☐ Other: _____

3. At what age does amblyopia typically begin? ☐ 0-1 yr ☐ 1-5 yrs ☐ 6-10 yrs ☐ 11-18 yrs ☐ Adulthood ☐ Unsure

4. Can untreated amblyopia affect long-term vision? ☐ Yes ☐ No ☐ Unsure

Section B: Knowledge of Causes, Symptoms, and Treatment 

1. Possible Causes of Amblyopia: (Select all) ☐ Eye misalignment ☐ Refractive errors ☐ Cataracts ☐ Genetic factors ☐ Unknown causes ☐ Other: _____

2. Symptoms of Amblyopia in Children: (Select all) ☐ Blurry vision ☐ Squinting ☐ Eye misalignment ☐ Difficulty in focusing ☐ Other: _____

3. Is amblyopia treatable if detected early? ☐ Yes ☐ No ☐ Unsure

4. Effective Treatments for Amblyopia: (Select all) ☐ Eye patching ☐ Corrective glasses ☐ Surgery ☐ Vision therapy ☐ Medications ☐ Other: _____

Section C: Personal Perceptions and Attitudes 

1. Do you think amblyopia should be part of routine child health screenings? ☐ Yes ☐ No ☐ Unsure

2. Have any of your children been screened for amblyopia? ☐ Yes ☐ No ☐ Unsure

## References

[1] AlJarallah OJ, AlFehaid MS, Alnadawi AA (2024). Knowledge and awareness regarding amblyopia among parents in Riyadh, Saudi Arabia: a cross-sectional study. Cureus.

[2] Abuallut II, Alameer KM, Abuageelah BM (2023). Parents' awareness, knowledge, and perception of amblyopia in children: a study in Jazan region, Saudi Arabia. Cureus.

[3] Alatawi A, Alali N, Alamrani A, Hashem F, Alhemaidi S, Alreshidi S, Albalawi H (2021). Amblyopia and routine eye exam in children: parent's perspective. Children (Basel).

[4] Alkalash SH, Alsayed HY, Alamshani TK (2023). Knowledge, attitude, and practice of parents regarding children's eye care in Al-Qunfudah Governorate, Saudi Arabia. Cureus.

[5] Pawar N, Ravindran M, Fathima A, Ramakrishnan K, Chakrabarthy S, Aparna K, Uduman MS (2023). Assessment of parental awareness about pediatric visual problems by knowledge-attitude-practice survey in South India. Indian J Ophthalmol.

[6] Sesma G, AlMairi T, Khashoggi H, Aljohar F, Khandekar R, Awad A (2022). Treatment outcome of occlusion for unilateral amblyopia in Saudi children 6-12 years old. Middle East Afr J Ophthalmol.

[7] Aldihan FA, Alamer NM, Alhejji A (2024). Knowledge and awareness of parents and the general population living in Riyadh about amblyopia. Cureus.

[8] Surrati AM, Almuwarraee SM, Mohammad RA, Almatrafi SA, Murshid SA, Khayat LI, Al-Habboubi HF (2022). Parents' awareness and perception of children's eye diseases in Madinah, Saudi Arabia: a cross-sectional study. Cureus.

[9] Al Mazrou A, Alsobaie NA, Abdulrahman AK, AlObaidan O (2020). Do Saudi parents have sufficient awareness of pediatric eye diseases in Riyadh?. Saudi J Ophthalmol.

[10] Alsaqr AM, Masmali AM (2019). The awareness of amblyopia among parents in Saudi Arabia. Ther Adv Ophthalmol.

[11] Alanazi SR, Alanazi HW, Alanazi WG (2024). Parents' knowledge and attitudes toward pediatric ophthalmic disorders in Saudi Arabia: a cross-sectional study. Pediatr Rep.

[12] Lam M, Suh D (2022). Screening, diagnosis, and treatment of pediatric ocular diseases. Children (Basel).

[13] Pineles SL, Repka MX, Velez FG, Yu F, Perez C, Sim D, Coleman AL (2022). Prevalence of pediatric eye disease in the optumlabs data warehouse. Ophthalmic Epidemiol.

[14] Kelkar J, Kelkar A, Thakur P, Jain HH, Kelkar S (2023). The epidemiology and disease pattern of pediatric ocular morbidities in Western India: the National Institute of Ophthalmology Amblyopia Study in Indian Paediatric Eyes (NIMBUS) study report 1. Indian J Ophthalmol.

[15] Almogbel AH, Al Shanbari N, Alibrahim IS, Alsaadi SS, Algarni HS, Alshanbari AS, Goweda R (2023). Parents' awareness and attitude toward pediatrics eye diseases in Makkah, Saudi Arabia: a cross-sectional study. Cureus.

[16] Alshammari LK, Alaradi LA, Alanazi AM, Almishali FF, Alabdullatif NH, Ali A (2024). Levels of awareness regarding pediatric eye diseases among Saudi parents from the Hail and Al-Qassim regions, Saudi Arabia. Cureus.

